# Effect of Light Intensity on Anthocyanin Synthesis Assessed Using Leaves of *Aglaonema commutatum*

**DOI:** 10.3390/genes16040375

**Published:** 2025-03-26

**Authors:** Xingxing Zhu, Canhang Wu, Junai Hui

**Affiliations:** College of Horticulture and Landscape Architecture, Zhongkai University of Agriculture and Engineering, Guangzhou 510225, China; xxzhu99@126.com (X.Z.); wu1075239927@126.com (C.W.)

**Keywords:** light intensity, *Aglaonema commutatum*, red leaf, gene expression

## Abstract

Background: Plant anthocyanins are a secondary metabolite widely distributed in the roots, stems, leaves, flowers, and fruits of plants, and their synthesis is significantly affected by light intensity. To investigate the synthesis of anthocyanins in *Aglaonema commutatum’s* leaves under different light intensities is essential. Methods: Using the commonly colored leaf *A. commutatum* variety ‘Emerald’ as the control group, and the red-leaf varieties ‘Red Ruyi’, ‘Angel’, and ‘Gilly Red’ as the experimental material, three light intensities were set: 254~368 μmol·m^−2^·s^−1^ (CK), 588~678 μmol·m^−2^·s^−1^ (T1), and 1125~1267 μmol·m^−2^·s^−1^ (T2). Results: The changes in anthocyanin content and anthocyanin-related gene expression in the leaves of *A. commutatum* with different leaf colors under different light intensities were studied. The results show that the anthocyanin content of *A. commutatum* leaves had a different trend compared to *A. commutatum* with increasing light intensity, and the appropriate light intensity could significantly promote anthocyanin synthesis after a certain time, and vice-versa. The anthocyanin content of CK and the T1 treatment was 1.14–3.72 times that of the T2 treatment; the photosensitive genes PHYB, CRY, and UVR8 were correlated with the anthocyanin synthesis of ‘Angel’ and ‘Gilly Red’. The anthocyanin structural genes *PAL*, *DFR*, and *ANS* were correlated with the anthocyanin synthesis of ‘Red Ruyi’, ‘Angel’, and ‘Gilly Red’. The anthocyanin transcription factor *bHLH* was strongly correlated with the anthocyanin synthesis of ‘Angel’. Conclusions: As a byproduct from *A. commutatum* leaves with ornamental value and potential economic value, this study was helpful to understand the potential mechanism of *A. commutatum’s* leaves where light intensity regulates anthocyanin synthesis and accumulation.

## 1. Introduction

Collectively known in the family Araceae, *A. commutatum* is a perennial evergreen flowering plant native to tropical Asian countries, including India, Thailand, Vietnam, Philippines, Malaysia, and Indonesia. Because of their bright and varied leaves and easy cultivation and management, they can be used as an excellent leaf viewing and fresh-cut ornamental plant, especially *A. commutatum*’s new variety with red leaves, which is becoming increasingly popular in the market. The content and proportion of pigment in the leaves are important factors in *A. commutatum*. Chlorophyll, carotenoids, and anthocyanin are the key factors determining *A. commutatum*’s leaf color, of which anthocyanin is the main pigment that shows red, purple, or blue [1].

As the main natural pigment, anthocyanins are flavonoids with special molecular structure and strong water solubility [2]. According to their molecular structure, anthocyanins can be divided into six classes: malva pigment, petunidin ligand, anthocyanidin, peonidin, delphinidin, and anthocyanins. Anthocyanins maintain a wide range of colors in plants, are the main color substances, and play an essential role in plants under stresses such as drought, low temperature, ultraviolet radiation, and pathogen infection [2,3,4,5]. In daily life, anthocyanins can be used as antioxidants to prevent cardiovascular disease, cancer, and some chronic diseases, and are also widely used in the food, medicine, and cosmetics industries [6]. The anthocyanin biosynthesis pathway is mainly divided into three stages. First, the precursor phenylalanine synthesizes 4-acyl-CoA via PAL (phenylalanine ammonlyase), C4H (*trans*-cinnamate 4-monooxygenase), and *4CL* (4-coumaric acid CoA Ligase 2) [7]. Second, 4-acyl-CoA and malonyl CoA are catalyzed by CHS (chalcone synthetase) to synthesize tetrahydroxyl chalcone, which is then isomerized by CHI (chalcone isomerization) to form the colorless compound trihydroxyl dihydroflavone, which is then catalyzed by *F3H* (dihydroflavone-3 hydroxylase) to synthesize dihydroflavones and dihydroflavonol [8]. Finally, *DFR* (dihydroflavonol-4-reductase) catalyzes the reduction of flavanones and dihydroflavonols to produce different colorless anthocyanins. These colorless anthocyanins are catalyzed by *ANS* (anthocyanin synthetase) to produce colored anthocyanins and UFGT (UDP-glucose-flavonoid 3-o-glucosyltransferase) to bind colored anthocyanins to glycosides, converting them to colored anthocyanins [9].

Light is one of the key environmental factors affecting the color of many plants. Light intensity, light quality, and the photoperiod affect the biosynthesis of anthocyanins, among which light intensity has the most significant effect. The expression of anthocyanin synthetase genes (*CHS*, *CHI*, *DFR*, *F3H*, *LDOX*, *F3′5′H*) and regulatory genes (*VvMYB30*, *VvbHLH79*, *VvbHLH121*) can be changed by the shading treatment in grape (*V. vifera* L.) to reduce the anthocyanin content. It was also found that the expression of structural genes (*LDOX*, *CHS*, *F3H*, *DFR*, *CHI*, *UFGT*) and regulatory genes (*MybA1*) were down-regulated under strong light, resulting in the decrease in the anthocyanin content [10,11]. The molecular mechanism of regulating anthocyanin biosynthesis by light intensity has been studied in many horticultural crops, such as blueberry [12] and pepper [13]. Therefore, studying the mechanism of influence of light intensity on anthocyanin synthesis is an important basis for improving anthocyanins in plants.

In *A. commutatum*, 26 anthocyanin biosynthesis structural genes and four key regulatory transcription factors were identified by transcriptome analysis, and it was further revealed that transcription factors (*AcMYB1* and *AcbHLH1*) interact with anthocyanin biosynthesis structural genes to regulate anthocyanin biosynthesis in *A. commutatum* [1,14]. However, the mechanism by which light intensity regulates anthocyanin biosynthesis from the leaves of *A. commutatum* has not been reported. Light intensity was taken as the investigation starting point, and the red-leaf rough-rib grass varieties *A. commutatum* ‘Red Ruyi’, ‘Angel’, and ‘Gilly Red’ and the evergreen-leaf *A. commutatum* variety ‘Emerald’ were used as the test materials. Taking into consideration the effect of different light intensities on the anthocyanin content and gene expression from *A. commutatum* leaves, the relationship between different light intensities and anthocyanin biosynthesis in *A. commutatum* leaves was investigated, so as to understand the environmental factors that affect *A. commutatum* leaf color change. We hope to provide a reference for the scientific conservation and management of *A. commutatum*.

## 2. Materials and Methods

### 2.1. Overview of the Test Site

The test site is located in the Baiyun campus, Zhongkai University of Agricultural and Engineering, Baiyun district, Guangzhou, with an altitude of 30 m, east longitude 113°26′, and north latitude 23°22′. It has a subtropical monsoon climate with a maximum temperature of 38 °C, a minimum temperature of 3 °C, and an annual average temperature of 22 °C. The annual effective accumulated temperature of ≥10 °C is 8000 °C, the annual precipitation is 1695.90 mm, the annual average relative humidity is 77%, the annual average sunshine duration is 1800 h, the frost-free period is as long as 11 and a half months, and the total solar radiation is 4500 MJ/m^2^.

### 2.2. Test Materials

We procured, from Xilin Horticulture Co., LTD., Guangzhou, China, *A. commutatum* as a disease-free and robust plant. Four varieties of *A. commutatum* aged 1.5 years old that were growing well and had basically the same growth (plant height of 30~40 cm) were selected, including *A. commutatum* with red leaves—*A. commutatum* ‘Red Ruyi’ (abbreviated as ‘Red Ruyi’), *A. commutatum* ‘Angel’ (abbreviated as ‘Angel’), and *A. commutatum* ‘Gilly Red’ (abbreviated as ‘Gilly Red’)—and a constant-colored leaf variety: *A. commutatum* ‘Emerald’ (abbreviated as ‘Emerald’). Samples were taken from 3 to 4 healthy mature leaves of *A. commutatum* ‘Emerald’ (from the tip of the plant).

### 2.3. Experimental Design

According to the actual situation of the pre-test and the cultivation of *A. commutatum*, three kinds of light intensity settings were set as follows: CK (heavy shade, 254–368 μmol·m^−2^·s^−1^), T1 (medium shade, 588–678 μmol·m^−2^·s^−1^), and T2 (natural light, 1125–1267 μmol·m^−2^·s^−1^). A LI-6800 portable photosynthesis system (LI-6800, LI-COR, Lincoln, NE, USA) was used to monitor the range of light intensity. A completely randomized block design was used, with 5 replicates per treatment and 1 replicate per 10 POTS per variety.

### 2.4. Sample Collection

According to the experimental protocol, *A. commutatum* plants with homogeneous growth should be selected for sample collection at different times after treatment. The 3 to 4 samples taken from healthy mature leaves of *A. commutatum* plants of the same size from the tip downward were randomly selected from each group; the leaf size was at a medium level on the whole plant and each biological cycle was repeated three times at each stage. The sample was placed in a foil-wrapped spiral tip bottom centrifuge tube, stored in liquid nitrogen, and returned to the laboratory for storage in a −80 °C ultra-low temperature refrigerator.

### 2.5. Measurement of Leaf Patch Area

At 0 d and 75 d, for the four varieties *A. commutatum*, a whiteboard was placed behind the fully grown functional leaf of *A. commutatum* and measurements were taken, and the green and white/red patch areas were determined by ImageJ 1.8.0 analysis.

### 2.6. Determination of Anthocyanin Content Index

The extraction method with 1% hydrochloric acid methanol was used. Three to four healthy samples from mature leaves with different leaf colors were taken from the tip of *A. commutatum* plants under each of the three light intensities. Leaf samples were accurately weighted (0.1 g) and put into a test tube containing 5 mL 1% hydrochloric acid and methanol (hydrochloric acid: methanol = 1:999,129 (*v*/*v*)) to extract the anthocyanins. After 24 h in the dark at 4 °C, the supernatant was collected and a UV-VIS spectrophotometer (TU-1810; Beijing Beifen Ruili Analytical Instrument Co., Ltd., Beijing, China) was used at 530 nm and 657 nm colorimetric wavelength.

### 2.7. Quantitative RT-qPCR

After 75 days of growth under different light conditions, the samples were subjected to RT-PCR experiments. The RT-qPCR method was conducted under the guidance of Taylor et al. [15]. Total RNA was isolated via an RNA extraction kit (Tsingke Bio Inc., Beijing, China). The concentration of each RNA sample was assessed using a ultramicrospectrophotometer (Aosheng, Nano-500, Hangzhou, China), and the integrity of the RNA was verified via gel electrophoresis. One microgram of isolated RNA was subsequently used to obtain first-strand cDNA via reverse transcription using the PrimeScript™ RT Kit with gDNA Eraser (Takara Bio Inc., Beijing, China). qRT-PCR analysis was performed using a fluorescence quantitative instrument (BIO RED CFX Connect Real-Time System, Shanghai, China). A two-step PCR amplification program was employed, including initial denaturation at 95 °C for 5 min, followed by 40 cycles of denaturation at 95 °C for 10 s and annealing at 60 °C for 30 s. Three biological replicates were performed for each program.

Fluorescent quantitative primers were validated by HieffTMqPCRSYBRGreenMasterMix (NoRox) (Yeasen, Shanghai, China). According to the full-length CDS sequence of the key synthetases in the anthocyanin biosynthesis pathway obtained by the transcriptome sequencing of *A. commutatum*, three anthocyanin structural genes, anthocyanin regulatory genes, and photosensitive genes were selected. The Primer Premier 5 software was used to design the specific primers, and the *A. commutatum* gene Actin was used as an internal reference. The primer sequences used are shown in Table 1 (all primers in this experiment were synthesized by Ruqi Biotechnology Co., Ltd. Guangzhou, China). The results were analyzed for relative quantification using the 2^−∆∆Ct^ method [16]. Origin 2022b was used for statistical mapping.

### 2.8. Data Processing

Excel 2019 and Origin 2022b software were used to organize the data and plot the experimental data. A one-way ANOVA was performed for all data by using the SPSS 26.0 software (SPSS Inc., Chicago, IL, USA) and the Duncan method (*p* < 0.05).

## 3. Results

### 3.1. Effect of Light Intensity on Leaf Patch Area from A. commutatum

**Figure 1:**
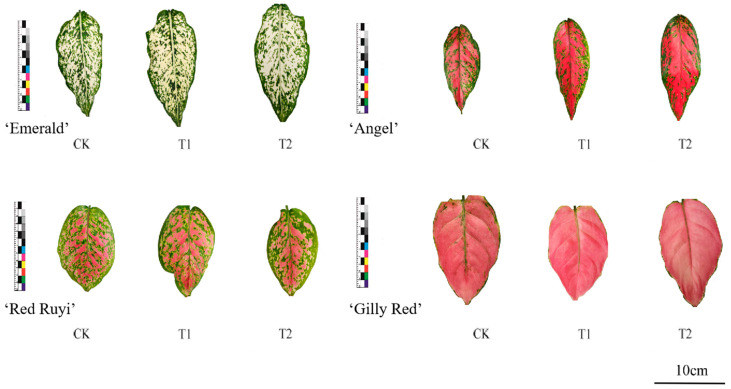
Leaf diagram of *A. commutatum* (0 d).

**Figure 2:**
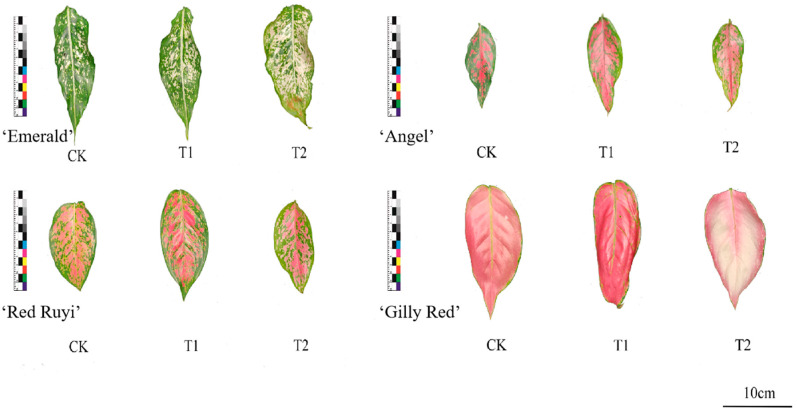
Leaf diagram of *A. commutatum* (75 d).

Leaf states of each *A. commutatum* variety at 0 d and 75 d are shown in Figure 1 and Figure 2. As can be seen from Table 2, the effect of different light intensities on the area proportion of the four *A. commutatum* color patches was not significant. At 75 days of treatment, ‘Emerald’ and ‘Gilly Red’ tend to increase first as the light intensity increases, while ‘Red Ruyi’ and ‘Angel’ tend to decrease and then increase as the light intensity increases. The value of ‘Emerald’ T2 decreased by 3.80% compared to that of CK, and increased by 4.20% compared to that on 0 d; ‘Red Ruyi’ T2 increased by 2.00% compared to CK in the same period. The value of ‘Angel’ T2 increased by 18.60% compared to that of CK; and ‘Gilly Red’ T2 decreased by 11.76% compared to CK.

When the area of white/red blocks was treated for 75 days, ‘Emerald’ and ‘Gilly Red’ showed an upward trend with the increase in light intensity, while ‘Red Ruyi’ and ‘Angel’ showed an upward trend and then a downward trend with the increase in light intensity. The value of ‘Emerald’ T2 increased by 4.20% compared to that of CK, and decreased by 3.80% compared to 0d. ‘Red Ruyi’ T2 decreased by 1.90% compared to CK. The value of ‘Angel’ T2 decreased by 14.00% compared to that of CK; and ‘Gilly Red’ T2 increased by 2.40% compared to CK in the same period. There was no significant difference between the effect of different light intensities on *A. commutatum* leaf patch color, possibly due to the treatment time being too short.

### 3.2. Effect of Light Intensity on Anthocyanin Content from A. commutatum Leaves

From the extraction of anthocyanins from *A. commutatum* leaves, it was found that the change trend of anthocyanins from the four different leaf colors of *A. commutatum* was different according to the light intensity treatment (Table 3). The anthocyanin content of ‘Emerald’ was the lowest among the four *A. commutatum* varieties, and the three *A. commutatum* treatment groups showed a tendency to increase and then decrease with time, and there was no significant difference between 15 d and 75 d under the three different light intensity treatments. But the anthocyanin content of the red-leaf *A. commutatum* variety showed different change trends with time according to the different light intensities: ‘Red Ruyi’ CK increases–decreases–increases, reaching the maximum value at 75 d, with an increase of 73.26% compared to 0 d. T1 decreased and then increased, and T2 increased and reached the maximum value at 75 d and 30 d, respectively, increasing by 15.05% and 50.19% compared to CK. ‘Angel’ CK presented a decreasing trend and then an increasing trend, reaching the lowest value at 30 d, decreasing by 69.00% compared to 0 d. T1 and T2 presented a decreasing trend with the passing of time, reaching the minimum value at 60 d and 75 d, respectively, decreasing by 21.09% and 71.12% compared to 0 d. The ‘Gilly Red’ CK presents an ascending and then descending trend, reaching the highest value at day 30 with an increase of 43.39% compared to day 0. T1 and T2 presented a descending–ascending–descending trend. T1 reached the maximum value at day 60 with an increase of 48.53% compared to CK in the same period; T2 reached the minimum value at day 75, with a decrease of 62.24% compared to CK in the same period.

The anthocyanin content of the red-leaf *A. commutatum* variety was significantly different during the whole process, and the anthocyanin content of CK *A. commutatum* and T1 *A. commutatum* was higher than that of T2, suggesting that bright light accelerates the consumption of anthocyanins from *A. commutatum* leaves, inhibiting anthocyanin biosynthesis and accumulation. The variety with the most obvious increase in anthocyanins was ‘Red Ruyi’. The anthocyanin content of the ‘Red Ruyi’, ‘Angel’, and ‘Gilly Red’ groups all began to differ significantly at 30 d, suggesting that the effect of different light intensities on *A. commutatum* leaves takes some time.

### 3.3. Effect of Light Intensity on the Expression of PHYB, CRY, and UVR8 Photosensitive Genes from A. commutatum Leaf

**Figure 3:**
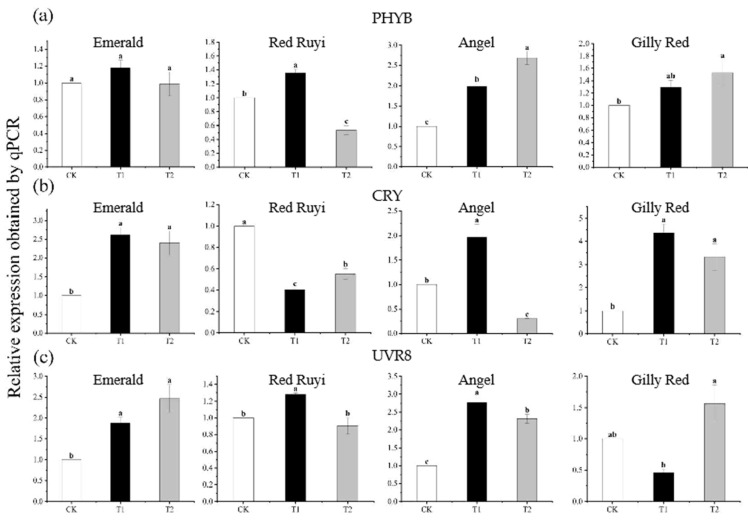
Effect of different light intensities on the expression of photosensitive genes in 3 cultivars of *A. commutatum*. After 75 days of growth under different light conditions, the samples were subjected to RT-PCR experiments. (**a**) is the expression of PHYB, (**b**) is the expression of CRY, (**c**) is the expression of UVR8. Note: Different lowercase letters indicate significant difference between varieties and treatments (*p* < 0.05).

The change trend of photosensitive gene expression from the leaves of the four different *A. commutatum* varieties under different light intensity treatments was different to some extent (Figure 3). The relative expressions of ‘Emerald’ CRY and UVR8 genes were significantly different with the increase in light intensity, and the relative expression of CRY genes in T1 and T2 was increased by 161.00% and 139.00% compared to that in CK, while UVR8 gene expression in T1 and T2 was increased by 87.60% and 147.00% compared to that in CK. The relative expressions of PHYB and CRY genes in ‘Red Ruyi’ increased and then decreased with the increase in light intensity. The relative expressions of PHYB and CRY genes increased 36.00% under the T1 treatment compared to CK, while CRY genes decreased 59.73 and 44.09% under the T1 and T2 treatments compared to CK. The relative expression of the ‘Angel’ PHYB gene showed a significant difference with the increase in light intensity, the relative expression of T2 increased 168.00% compared to CK, CRY and UVR8 genes increased and then decreased with the increase in light intensity, and the relative expression of T1 increased 97.00% and 177.00% compared to CK. Compared to CK, the relative expression of CRY in T2 decreased by 69.63%. The expression level of the ‘Gilly Red’ CRY gene increased and then decreased with the increase in light intensity, with a significant difference between treatments. The relative expression level of T1 increased by 337.00% and that of T2 increased by 232.00% compared to that of CK.

### 3.4. Effect of Light Intensity on Expression of PAL, DFR, and ANS of Leaf Anthocyanin Structural Genes from A. commutatum

**Figure 4:**
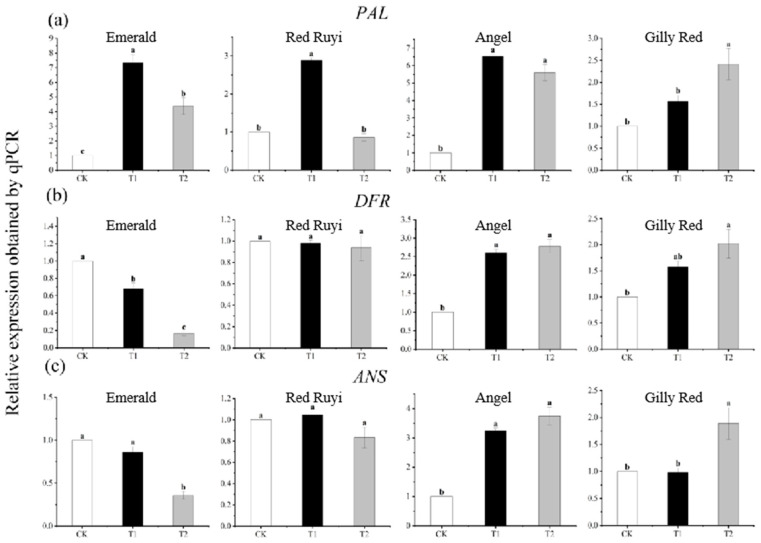
Expression of three anthocyanin structural genes (*PAL*, *DFR*, *ANS*). (**a**) is the expression of *PAL*, (**b**) is the expression of *DFR*, (**c**) is the expression of *ANS*. Note: Different lowercase letters indicate significant difference between varieties and treatments (*p* < 0.05). After 75 days of growth under different light conditions, the samples were subjected to RT-PCR experiments.

The expression of anthocyanin structural genes from *A. commutatum* differs to different degrees with different light intensities (Figure 4), among which the relative expressions of ‘Emerald’ *PAL*, *DFR*, and *ANS* genes increased and then decreased with the increase in light intensity, and *PAL* expression was the highest in T1. The lowest relative expressions of *DFR* and *ANS* in T2 increased by 633.00% and decreased by 83.58% and 64.28% compared to CK, respectively. The relative expression level of the ‘Red Ruyi’ *PAL* gene increased and then decreased with the increase in light intensity, and there was a significant difference between treatments. The relative expression level of T1 was the highest, which increased by 187.00% compared to CK. The relative expression level of the ‘Angel’ *PAL* gene increased and then decreased with the increase in light intensity, and there was a significant difference between treatments. Compared to CK, the relative expression levels of T1 and T2 increased by 552.00% and 460.00%, respectively, and the relative expression levels of the *DFR* and *ANS* genes increased significantly with the increase in light intensity. Compared to CK, the relative expression level in T2 increased by 178.21% and 275.26%. The relative expression levels of the ‘Gilly Red’ *PAL*, *DFR*, and *ANS* genes were significantly different with the increase in light intensity, and the relative expression levels in T2 increased by 141.00%, 102.00%, and 88.76% compared to CK.

### 3.5. Effect of Light Intensity on the Expression of MYB, bHLH, and WDR from Leaf Anthocyanin Regulatory Genes of A. commutatum

**Figure 5:**
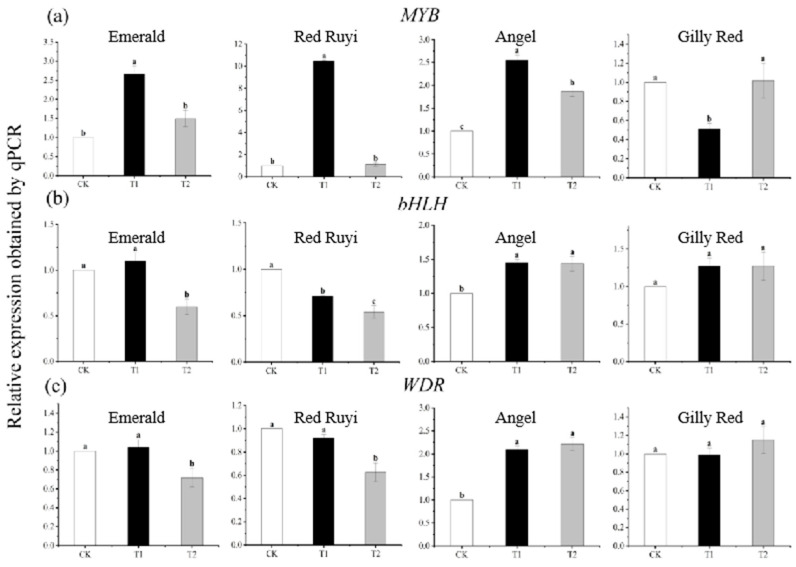
Expression of three transcription factors (*MYB*, *bHLH*, *WDR*). (**a**) is the expression of *MYB*, (**b**) is the expression of *bHLH*, (**c**) is the expression of *WDR*. Note: Different lowercase letters indicate significant difference between varieties and treatments (*p* < 0.05). After 75 days of growth under different light conditions, the samples were subjected to RT-PCR experiments.

The expression of anthocyanin structural genes was also regulated by transcription factors, and there was a significant difference in the expression of *A. commutatum* regulatory genes after different light intensity treatments (Figure 5). The relative expression levels of the ‘Emerald’, ‘Red Ruyi’, and ‘Angel’ *MYB* genes increased and then decreased with the increase in light intensity, with significant differences among treatments, and the highest expression levels were found in T1, which increased by 165.32%, 94.16%, and 154.00% compared to CK, respectively. The relative expression of the *bHLH* gene decreased with the increase in light intensity in ‘Red Ruyi’, decreased by 46.13% compared to CK in ‘Gilly Red’, and increased by 27.14% compared to CK in ‘Gilly Red’. In ‘Angel’, the relative expression of the *WDR* gene increased with the increase in light intensity, and in T2, it increased by 121.54% compared to that in CK, while in ‘Red Ruyi’, the trend was opposite and the amplitude decreased, and in T2, it decreased by 37.41% compared to that in CK. 

### 3.6. Correlation Between Anthocyanin Content from A. commutatum Leaves and Photoreceptor Genes, Anthocyanin Structural Genes, and Anthocyanin Regulatory Genes

**Figure 6:**
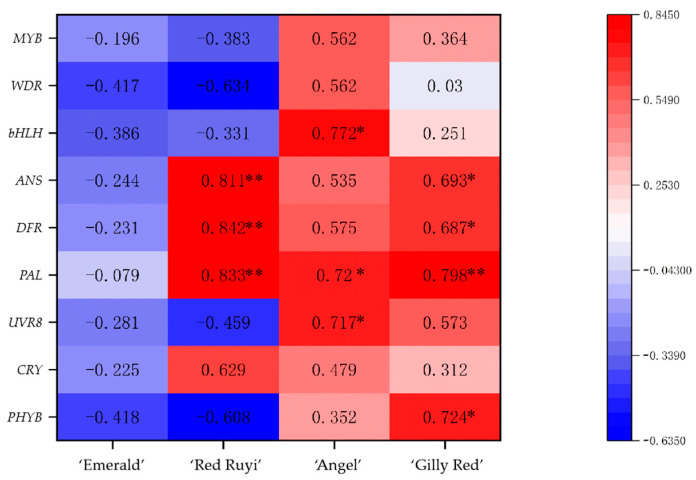
Association of anthocyanin content with photoreceptor genes, anthocyanin structural genes, and anthocyanin regulatory genes in *A. commutatum*. Note: “*” shows significant correlation at the *p* < 0.05 probability level, “**” shows significant correlation at the *p* < 0.01 probability level.

There are different correlations between the anthocyanin content of the four kinds of *A. commutatum* leaves and the genes involved in anthocyanin synthesis (Figure 6). The relative expressions of the three photoreceptor genes of ‘Emerald’ increased with the increase in light intensity, inducing *PAL* and *MYB* gene synthesis, while decreasing the relative expressions of *DFR*, *ANS*, *bHLH*, and *WDR* genes. There was no significant correlation between ‘Emerald’ anthocyanin synthesis and photoreceptor genes, and between anthocyanin structural genes and regulatory genes. With the increase in light intensity, the relative expression levels of the ‘Red Ruyi’ PHYB, UVR8, *ANS*, *PAL*, *DFR*, and *MYB* genes increased, but decreased the relative expression levels of the *bHLH*, *WDR*, and CRY genes increased. Three anthocyanin structure genes, *PAL*, *DFR* and *ANS*, were strongly correlated with anthocyanin synthesis in ‘Red Ruyi’. With the increase in light intensity, the relative expression levels of ‘Angel’ PHYB, UVR8, *ANS*, *PAL*, *MYB*, *bHLH*, and *WDR* increased, while the relative expression levels of CRY decreased. The anthocyanin synthesis of ‘Angel’ was related to the expression of the photosensitive gene UVR8, structural gene *PAL*, and regulatory gene *bHLH*. With the increase in light intensity, the relative expression levels of three kinds of photosensitive genes, anthocyanin structural genes, and regulatory genes in ‘Gilly Red’ increased, and anthocyanin synthesis in ‘Gilly Red’ was related to the expression of the photic gene PHYB and the expression of three kinds of structural genes, *PAL*, *DFR*, and *ANS*.

## 4. Discussion

### 4.1. Effect of Light Intensity on Anthocyanin Content in Plants

Various studies have shown that anthocyanin accumulation is affected by environmental factors, especially light intensity [17]. In general, a higher light intensity is required to induce anthocyanin synthesis, and the anthocyanin content in plant leaves is related to the light level. The leaf color change in red-leaf plants can be quantitatively compared from the content of anthocyanins. Shading changes the light intensity of the local environment, thus affecting the content and proportion of chlorophyll, carotenoids, and anthocyanins, then affecting the color of the leaves of color-leaf plants. In grapes, anthocyanin accumulation was increased by strong light, but inhibited by shade [17]. In this study, the anthocyanin content of *A. commutatum* leaf increased and then decreased with the increase in light intensity. Of the four kinds of *A. commutatum*, the anthocyanin content of *A. commutatum* ‘Emerald’ leaves was the lowest, and there was no significant difference between the three different treatments of light intensity. From red-leaf *A. commutatum* ‘Red Ruyi’, ‘Angel’, and ‘Gilly Red’, the highest anthocyanin content was from T1 *A. commutatum* ‘Red Ruyi’, ‘Angel’, and ‘Gilly Red’, then from CK *A. commutatum*, and T2 was the lowest. That is, the biosynthesis and accumulation of anthocyanins from red-leaf *A. commutatum* varieties at low light was relatively large, whereas the bright light consumes anthocyanin storage. It is inferred that the positive regulation of anthocyanin content of commutatum leaves is in the range of from 588 to 678 μmol·m^−2^·s^−1^.

### 4.2. Effects of Photoreceptors on Plants

Light is an important source of energy for plants, but also, as an environmental signal throughout the whole life cycle of plants, it can affect the shape of plants and adaptability to the environment. Light signals can be divided into light intensity, light quality, photoperiod, and direction of light, perceived by different photoreceptors. At present, there are at least four kinds of photoreceptors in the plant kingdom according to their absorption spectra: red light/far-red light receptor phytochrome (PHY), blue light receptor CRYptochrome (CRY), phototropic protein (PHOT), and UV-B receptor UVR8 (UV Resistance locus 8) [18]. Phytochrome (PHY) mainly absorbs red light/far-red light (wavelength 600~750 nm). As a class of well-studied photoreceptors in higher plants, phytochromes can sense red/far-red light, and studies have also shown that they can promote anthocyanin accumulation [19]. In this study, the anthocyanin content from the leaves of *A. commutatum* ‘Angel’ and ‘Gilly Red’ was positively correlated with PHYB as the light intensity increased. Cryptochrome (CRY), or blue/UV-A receptor, is a receptor that senses light in the blue and near-ultraviolet (330–390 nm) regions; it can regulate the growth and development of plants [20]. Phototropin (PHOT) mainly absorbs blue light/UV-A (380–500 nm) [21]. UVR8 (UV Resistance Locus 8), as a specific photoreceptor of UV-B, mainly absorbs light in the UV-B region (wavelength ranging from 282 to 320 nm), and it can rapidly respond to the stimulation of UV-B light. And it regulates the growth and development of plants by regulating DNA repair, inhibiting growth, and regulating photosynthesis. In addition, UVR8 receptors can also regulate the photoprotective mechanism of plants, thereby protecting plants from excessive UV radiation damage [21]. Studies have shown that UVR8 receptors are found in lychees, apples, and in the ripening process of fruits in plants such as *Solanum melongena* [22]. ‘Emerald’ displayed an attenuated responsiveness to light intensity variations (UVR8 upregulation in Figure 3c failed to activate downstream anthocyanin biosynthesis pathways), whereas red-leaf cultivars exhibited stronger coupling between light signal transduction and anthocyanin synthesis. This discrepancy may stem from genetic background differences (e.g., regulatory element variations). The quantitative PCR relative expression levels of the *PHYB*, *CRY*, and *UVR8* genes from *A. commutatum* of different varieties were analyzed under different light intensities, and the results show that the expression of *A. commutatum* was different. With the increase in light intensity, the relative expressions of the ‘Angel’ and ‘Gilly Red’ *PHYB* genes in T2 were increased by 168.00% and 52.00% compared to those of CK, respectively. The increase in light intensity decreased the expression of the ‘Red Ruyi’ *CRY* gene and induced the expression of the ‘Emerald’ and ‘Gilly Red’ *CRY* genes to increase by 161.00% and 337.00%, respectively, in T1 compared to CK. The increase in light intensity induced the expression of the ‘Angel’ *UVR8* gene in T1 to increase by 177.00% compared to CK, and the expression of the ‘Emerald’ and ‘Gilly Red’ *UVR8* gene in T2 increased by 147.00% and 56.60% compared to that of CK, respectively.

### 4.3. Expression of Genes Related to Anthocyanin Synthesis in Plants

Anthocyanin synthesis, transport, and accumulation are affected by various genes in the synthetic pathway. Ahn et al. found that the production of red leaves in the red variety of *Zoysia japonica Steud*. was caused by the increased expression of *DFR* and *ANS*, and was also specifically and synergistically regulated by a variety of transcription factors [23]. At present, the three most studied and important types of transcription factors in the anthocyanin synthesis pathway are the *MYB* transcription factor family, *bHLH* transcription factor family, and *WDR* transcription factor family [24]. Anthocyanin biosynthesis requires the coordinated regulation of multiple gene categories (structural genes, light signaling components, and transcription factors). While certain genes (e.g., *PAL*, *MYB*) exhibited elevated expression in ‘Emerald’ (Figure 4a and Figure 5a), the downstream critical structural genes (*DFR* and *ANS*) showed a significantly lower expression compared to those of the red-leaf cultivars (Figure 4b,c). This bottleneck likely disrupts anthocyanin biosynthesis, ultimately maintaining the green-leaf phenotype. In this experiment, analysis of the gene expression related to the anthocyanin synthesis pathway showed that light was affected by the expressions of *PAL*, *DFR*, and *ANS* and regulatory genes *MYB*, *bHLH*, and *WDR* involved in the four structural genes of *A. commutatum* leaf anthocyanin synthesis. Studies have shown that the apple *MdMYB1* transcription factor is a light-induced *R2R3-MYB* transcription factor, and light induces the expression of *MdMYB1*, thereby inducing anthocyanin synthesis [25]. In addition, relevant studies have revealed the key regulatory role of a second transcription factor, *bHLH*, which was highly correlated with the transcription factor *MYB*. During the ripening process of *Garcinia Mangstana* L. fruit from green to purple, the transcription factor *Gm MYB10* changed the most, and *Gm MYB10* and *AtbHLH2* were transferred into tobacco, which effectively activated the *GmDFR* and *AtDFR* promoters [26]. In *Chrysanthemum morifolium ramat*, Xiang et al. found that *CmMYB6* and *CmbHLH2* formed a binary complex through physical interaction, which regulated the expression of *CmDFR* during the biosynthesis of chrysanthemum anthocyanins [27]. Studies have confirmed that the *MYB* transcription factor alone or the combination of *WD40* gene *TTG1*, *bHLH*, and *MYB* transcription complex can regulate the anthocyanin biosynthesis pathway [28,29]. In this study, the relative expression levels of the leaf anthocyanin structural genes and regulatory genes from *A. commutatum* at different light intensities were analyzed. Previous studies have also found that *AcMYB1* and *AcbHLH1* interact with *A. commutatum* to regulate the biosynthesis of anthocyanins [14]. It can be inferred that there is a certain relationship between the structural genes of *A. commutatum* leaves and the regulatory genes.

## 5. Conclusions

The anthocyanin content of *A. commutatum* leaves decreased significantly under high light. Light intensity affects anthocyanin synthesis by regulating structural genes from the *A. commutatum* leaf anthocyanin synthesis pathway and regulatory gene expression. Differential expressions of genes involved in anthocyanin biosynthesis were analyzed by qRT-PCR. The correlation analysis showed that light-sensitive genes, structural genes, and anthocyanin regulatory genes of the *A. commutatum* leaves of three red-leaf varieties are significantly affected by the anthocyanin content. This study provides reference for the scientific conservation and management of *A. commutatum* cultivation, and is also helpful to understand the effect of light environment on anthocyanin synthesis and accumulation in *A. commutatum* leaves.

## Figures and Tables

**Table 1:** Fluorescent quantitative PCR primers.

Primer Names	Upstream Primer Sequence	Downstream Primer Sequence
Actin	AGAGCTATGAGCTGCCTGAC	CATCCCGATGAGAGACGGTT
PHYB	AATGTGACAGACAGTTCAGTAGGAA	GTGGTGAGGTACAATACATCAGAGA
CRY	TCAACTTACGTCAGGGTGATGTT	GGAGGATATCATAGGTGCCAAC
UVR8	AGCCGACCATATCAGGAACC	CAAGGAGACATCGGCATCCA
PAL	AACATCCTGGCGGTACTGG	GCCTCTTGGCCATCTTTACG
DFR	TCGTGGGAGGAGCAGATGTATC	CCGCACTACTCCATCCTGAAC
ANS	CTATTACGAGGGCCAGTGGG	CTCTTGTACAGCCCGTTGCT
bHLH	TGCCTTAAAGCTGGTGGTTCA	TTGGGTGCTTCAGAGGGTTG
WDR	CCCTCGACAACACCCTGAAA	TGATCCTCAGAACCACTGACA
MYB	ACTACTGGAACTCCCACCTCA	GGTCCATGATTACGCGAGCA

**Table 2:** Effect of light intensity on the proportion of leaf patch areas.

Time/d	*A. commutatum*	Treatment	Green Color Block Area Proportion/%	White/Red Color Block Area Proportion/%
0	‘Emerald’	CK	45.00 ± 2.00 a	55.00 ± 2.00 a
		T1	44.00 ± 1.00 a	56.00 ± 1.00 a
		T2	48.00 ± 5.00 a	52.00 ± 5.00 a
0	‘Red Ruyi’	CK	44.00 ± 7.00 a	56.00 ± 7.00 a
		T1	44.00 ± 1.00 a	56.00 ± 1.00 a
		T2	46.00 ± 8.00 a	54.00 ± 8.00 a
0	‘Angel’	CK	45.00 ± 5.00 a	55.00 ± 5.00 a
		T1	40.00 ± 3.00 a	60.00 ± 3.00 a
		T2	42.00 ± 4.00 a	58.00 ± 4.00 a
0	‘Gilly Red’	CK	10.00 ± 2.00 a	90.00 ± 2.00 a
		T1	12.00 ± 3.00 a	88.00 ± 3.00 a
		T2	8.00 ± 1.00 a	92.00 ± 1.00 a
75	‘Emerald’	CK	52.00 ± 4.00 a	48.00 ± 4.00 a
		T1	50.00 ± 1.00 a	50.00 ± 1.00 a
		T2	50.00 ± 2.00 a	50.00 ± 2.00 a
75	‘Red Ruyi’	CK	49.00 ± 6.00 a	51.00 ± 6.00 a
		T1	42.00 ± 8.00 a	58.00 ± 8.00 a
		T2	50.00 ± 4.00 a	50.00 ± 4.00 a
75	‘Angel’	CK	43.00 ± 3.00 a	57.00 ± 3.00 a
		T1	41.00 ± 4.00 a	59.00 ± 4.00 a
		T2	51.00 ± 3.00 a	49.00 ± 3.00 a
75	‘Gilly Red’	CK	17.00 ± 1.00 a	83.00 ± 1.00 a
		T1	16.00 ± 5.00 a	84.00 ± 5.00 a
		T2	15.00 ± 3.00 a	85.00 ± 3.00 a

Note: Different lowercase letters indicate significant differences between cultivars and treatments (*p* < 0.05).

**Table 3:** Effect of light intensities on the anthocyanins of *A. commutatum* (pigment unit).

*A. commutatum*	Treatment	Time/d					
		0	15	30	45	60	75
‘Emerald’	CK	1.15 ± 0.13 a	1.06 ± 0.07 a	1.35 ± 0.02 a	1.74 ± 0.02 a	2.05 ± 0.12 a	1.80 ± 0.05 a
	T1	1.11 ± 0.08 a	1.33 ± 0.06 a	1.03 ± 0.26 a	1.62 ± 0.12 a	2.04 ± 0.21 a	1.63 ± 0.08 a
	T2	1.54 ± 0.10 a	1.02 ± 0.14 a	1.47 ± 0.09 a	1.88 ± 0.11 a	1.68 ± 0.01 a	1.64 ± 0.05 a
‘Red Ruyi’	CK	3.03 ± 0.71 a	4.18 ± 0.12 a	2.67 ± 0.33 b	2.97 ± 0.15 b	3.14 ± 0.13 b	5.25 ± 0.16 a
	T1	3.28 ± 0.27 a	2.77 ± 0.19 b	3.51 ± 0.13 a	4.99 ± 0.25 a	5.15 ± 0.35 a	6.04 ± 0.44 a
	T2	3.08 ± 0.90 a	2.60 ± 0.13 b	4.01 ± 0.12 a	2.93 ± 0.19 b	3.22 ± 0.29 b	3.68 ± 0.37 b
‘Angel’	CK	11.84 ± 3.10 a	9.52 ± 0.28 a	3.67 ± 0.33 b	4.33 ± 0.20 ab	5.66 ± 0.28 a	7.41 ± 0.44 a
	T1	7.68 ± 0.76 a	7.31 ± 0.73 b	7.38 ± 0.81 a	6.22 ± 1.27 a	6.06 ± 0.41 a	6.49 ± 0.40 a
	T2	9.54 ± 2.19 a	10.32 ± 0.56 a	5.08 ± 0.65 b	3.10 ± 0.27 b	4.17 ± 0.33 b	2.75 ± 0.09 b
‘Gilly Red’	CK	4.24 ± 1.13 a	3.37 ± 0.18 a	6.08 ± 0.84 a	4.80 ± 0.05 b	5.11 ± 0.14 b	4.37 ± 0.24 b
	T1	5.50 ± 0.14 a	4.30 ± 0.34 a	5.64 ± 0.44 a	7.43 ± 0.34 a	7.59 ± 0.28 a	6.13 ± 0.26 a
	T2	4.65 ± 0.34 a	3.61 ± 0.38 a	2.97 ± 0.14 b	2.67 ± 0.16 c	3.66 ± 0.39 c	1.65 ± 0.04 c

Note: Note: Different lowercase letters indicate significant differences between varieties and treatments (*p* < 0.05). The same applies below.

## Data Availability

The raw data supporting the conclusions of this article will be made available by the authors upon request.
